# Assessing the Predictive Factors for Bleeding in Esophageal Variceal Disease: A Systematic Review

**DOI:** 10.7759/cureus.48954

**Published:** 2023-11-17

**Authors:** Camila Guinazu, Adolfo Fernández Muñoz, Maria D Maldonado, Jeffry A De La Cruz, Domenica Herrera, Victor Sebastian Arruarana, Ernesto Calderon Martinez

**Affiliations:** 1 Internal Medicine, Universidad del Salvador, Buenos Aires, ARG; 2 Cardiovascular Medicine, Queen Elizabeth Hospital, Bridgetown, BRB; 3 Cardiovascular Medicine, Universidad de Ciencias Médicas - Santiago de Cuba, Santiago de Cuba, CUB; 4 Medicine, Faculty of Medicine, Universidad Nacional de Córdoba, Cordoba, ARG; 5 Medicine, Universidad Tecnológica de Santiago (UTESA), Santiago de los Caballeros, DOM; 6 Medicine, Pontificia Universidad Católica del Ecuador, Quito, ECU; 7 Internal Medicine, Brookdale University Hospital Medical Center, New York, USA; 8 Biomedical Informatics, Universidad Nacional Autónoma de México, Mexico City, MEX

**Keywords:** systematic review, internal medicine, risk of bleeding, gastroenterology, esophageal varcices

## Abstract

Esophageal varices, dilated submucosal distal esophageal veins, are a common source of upper gastrointestinal bleeding in patients with portal hypertension. This review aims to comprehensively assess predictive factors for both the first occurrence and subsequent risk of esophageal variceal bleeding. A systematic search was conducted in PubMed/MEDLINE (Medical Literature Analysis and Retrieval System Online) and Cochrane databases. A total of 33 studies were selected using rigorous inclusion and exclusion criteria. The risk of bias was assessed using the Newcastle-Ottawa Scale. Several predictive factors were identified for esophageal variceal bleeding, including the Child-Pugh score, Fibrosis Index, specific endoscopic findings, ultrasound parameters, portal vein diameter, presence and size of collaterals, CT scan findings, ascites, platelet counts, coagulation parameters, albumin levels, Von Willebrand Factor, bilirubin levels, diabetes mellitus, and the use of b-blocking agents in primary prophylaxis. The findings of this systematic review shed light on multiple potential predictive factors for esophageal variceal bleeding. Endoscopic findings were found to be reliable predictors. Additionally, ultrasound parameters showed associations with bleeding risk. This systematic review identifies multiple potential predictive factors for esophageal variceal bleeding in patients with portal hypertension. While certain factors exhibit strong predictive capabilities, further research is needed to refine and validate these findings, considering potential limitations and biases. This study serves as a critical resource for bridging knowledge gaps in this field.

## Introduction and background

Esophageal varices are dilated submucosal distal esophageal veins connecting the portal and systemic circulations [​1]. The response of the body to the increased venous pressure is the development of collaterals. These portosystemic collaterals divert blood from the portal venous system to the inferior and superior vena cava. At the same time, one important system is the gastroesophageal collaterals that drain into the azygos vein and lead to the development of esophageal varices. When these varices get enlarged, they rupture, causing severe hemorrhage​ [1]. ​   

Variceal hemorrhage represents approximately 70% of all upper gastrointestinal bleeding (UGB) episodes in patients with portal hypertension. In a cross-sectional study conducted in the United States from 2005 to 2014, a total of 348,958 (34,895 per year) patients were hospitalized with esophageal variceal bleeding​ [2]; furthermore, the mortality rate in patients with acute variceal bleeding is as high as 12-22% ​[3].​   

Predicting the occurrence of esophageal variceal bleeding and effectively stratifying individuals susceptible to this condition holds the promise of significantly reducing hospitalizations and mortality rates. Several scoring systems have been developed for the assessment of patients with UGB to predict clinical outcomes. The most frequently used are the Rockfall score, the Glasgow-Blatchford score (GBS), and AIMS65 (albumin, international normalized ratio (INR), mental status, systolic blood pressure, age >65 years) [4]. However, these scoring systems are limited to assessing post-bleed risk [5], leaving​ a critical gap in predicting the initial bleeding episode. This study aims to comprehensively assess predictive factors for both the first occurrence and the subsequent risk of esophageal variceal bleeding.   

## Review

Methods

Search Strategy

Pubmed/MEDLINE (Medical Literature Analysis and Retrieval System Online and Cochrane databases were searched using Medical Subject Headings (MeSH) terms and free-text terms (Tables 1, 2) on September 19, 2023.  This review followed the Preferred Reporting Items for Systematic Reviews and Meta-Analyses (PRISMA) guidelines in the selection of articles for the systematic review and meta-analysis [6].  This rigorous approach resulted in a homogeneous dataset, allowing for more accurate and reliable results.  

**Table 1:** Search terms on PubMed/MEDLINE MEDLINE: Medical Literature Analysis and Retrieval System Online

Search terms	Results
((Esophageal Varices[MeSH Terms]) OR (Variceal Bleeding[MeSH Terms]) OR (Esophageal Hemorrhage[MeSH Terms]) OR (Esophageal Variceal Disease[Title/Abstract])) AND ((risk assessment[MeSH Terms]) OR (Predictive Factors[MeSH Terms]) OR (Bleeding Risk[Title/Abstract]) OR (Hemorrhage Risk[Title/Abstract]) OR (Predictors[Title/Abstract]) OR (Risk Factors[Title/Abstract]))	1414

**Table 2:** Search on Cochrane

Search terms	Results
#1 MeSH descriptor: [Esophageal and Gastric Varices] explode all trees	1031
#2 (Esophageal Variceal Disease):ti,ab,kw	309
#3 MeSH descriptor: [Risk Assessment] explode all trees	13634
#4 (Bleeding Risk):ti,ab,kw	15651
#5 (Hemorrhage Risk):ti,ab,kw	10756
#6 (Predictors):ti,ab,kw	23474
#7 (Risk factors):ti,ab,kw	90879
#8 (#1 OR #2) AND (#3 OR #4 OR #5 OR #6 OR #7)	326

Inclusion and Exclusion Criteria

The review included studies that satisfied the search terms, were published between 2003 and 2023, and were written in either Spanish or English. A rigorous exclusion criterion was applied to ensure the quality and relevance of the studies included in the analysis. Studies with participants who were not diagnosed with portal hypertension were excluded. In addition, studies that were not available in full text or could not be obtained through interlibrary loans were excluded. The detailed inclusion and exclusion criteria in different categories are detailed below.

Types of studies: Observational studies, cohort studies, and case-control studies were included. Case reports, case series, cross-sectional studies, dissertations, book chapters, protocol articles, reviews, news articles, conference abstracts, letters to the editor, editorials, and comment publications were excluded, as these may not provide the depth of data required for our analysis. Additionally, studies lacking a clear operationalization description, duplicates, and those where necessary data couldn’t be obtained or where we didn’t receive a response from the original author were also excluded, ensuring the precision and reliability of the studies included in our systematic review.   

Types of participants: Studies with adult participants (aged ≥ 18 years) of all genders diagnosed with portal hypertension, regardless of its etiology, were included. Patients with confirmed esophageal varices through relevant diagnostic procedures such as endoscopy, imaging, or clinical assessment. We included patients with or without a history of bleeding secondary to esophageal varices. On the other hand, patients without a diagnosis of portal hypertension, those experiencing UGB unrelated to esophageal varices (including gastric varices, esophageal trauma, gastric mucosal lesions, benign or malignant tumors of the stomach and esophagus, and peptic ulcer disease) were excluded. Studies pertaining to UGB secondary to coagulopathy or anticoagulant therapy and pediatric populations (individuals under 18 years of age) were also excluded. These refined criteria aim to enhance the study’s focus and ensure the selection of appropriate participants for investigating predictive factors associated with the risk of bleeding in esophageal variceal disease.  

Types of intervention: In this review, there was no specified type of intervention outlined within the methodology. Unlike some research studies that focus on evaluating specific interventions or treatment, the primary aim of the current review was to identify and assess predictive factors associated with the risk of bleeding in esophageal variceal disease like portal hypertension severity, variceal size and location, liver function, coagulation profile, and other relevant parameters. The goal was to comprehensively understand and delineate these factors that could aid in early interventions, better prognosis, and management strategies with a posterior reduction in mortality and number of hospitalizations. This allows for a broad investigation into clinical and diagnostic factors without being confined to a specific intervention or treatment modality.  

Outcomes: Studies that focused on identifying predictive factors associated with bleeding risk in esophageal variceal disease were included. Participants should have undergone an assessment of predictive factors for bleeding in esophageal variceal disease; participants with incomplete or missing data on this matter will be excluded. Studies that aimed to establish correlations between these factors and the occurrence of bleeding in esophageal varices were included.

*Selection of Studies/Data Extraction *  

Following an initial screening based on the title and abstract, two reviewers (MMM and JACP) independently selected trials for inclusion in this review using predetermined inclusion and exclusion criteria.  This search was performed by using Rayyan​ [7]​ to extract relevant data; duplicates were filtered. We resolved any disagreements about the inclusion of studies by consensus and consultation with a third review author (CG).

Data Evaluation/Assessment of Risk of Bias

Data were evaluated using the criteria outlined in Cochrane. To assess the quality of studies included in the systematic review, for case-control and cohort studies, we employed the Newcastle-Ottawa Scale (NOS)​ [8]​ to assess the risk of bias. Two independent blinded reviewers (CG and AFM) evaluated the risk of bias in each study, considering the specific criteria and guidelines provided by the respective tools. Any discrepancies between the reviewers were resolved through discussion or by consulting with a third, blinded reviewer as needed (ECM).

The methodological components of the case-control and cohort studies were assessed as having a low, high, or unclear risk of bias in accordance with the Cochrane Handbook for Systematic Reviews of Interventions [9], and the NOS guidelines [8], respectively. Details of any down-or-upgrading of the quality of evidence were presented in the summary of the findings table, providing transparency and explanations for the assessment of bias in each study included.

Results

*Study Identification and Selection * 

**Figure 1:**
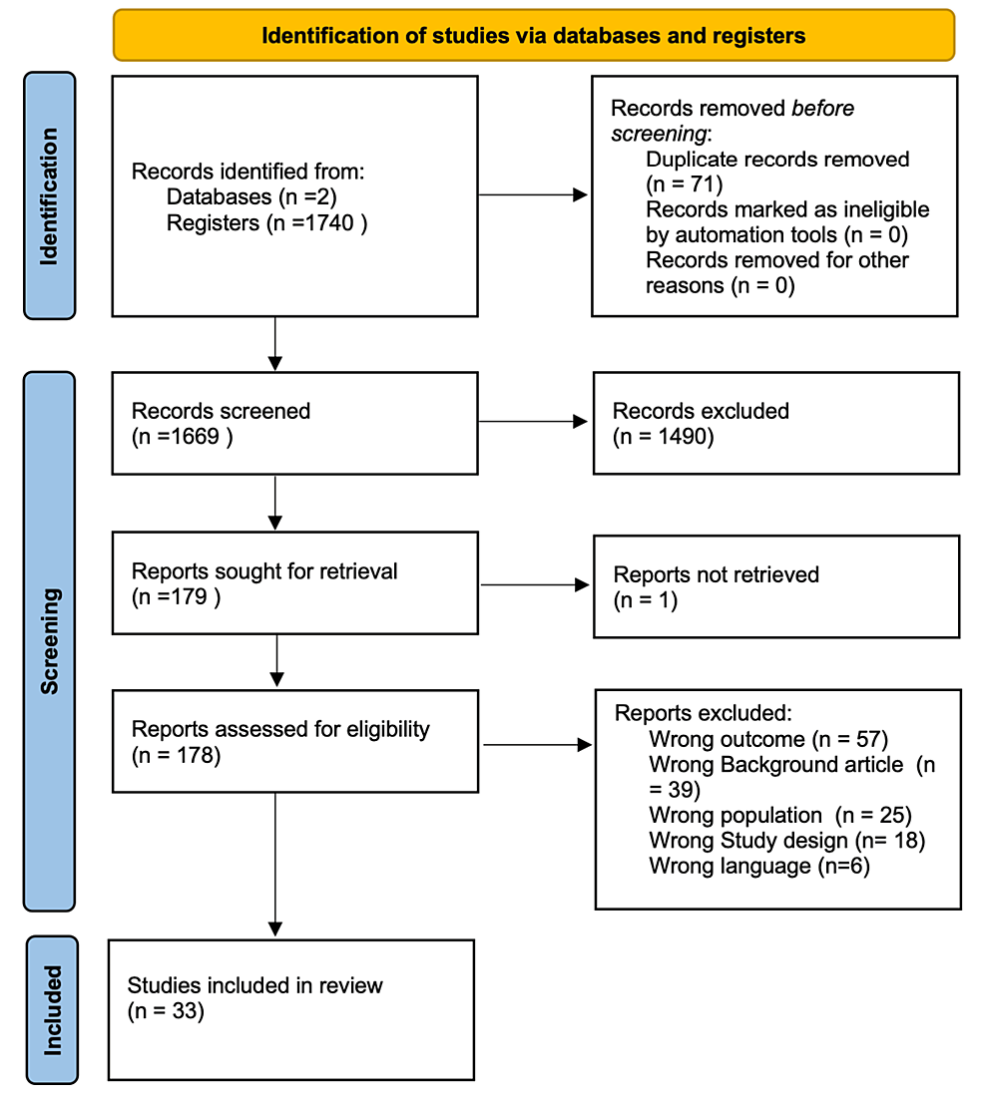
PRISMA flow diagram PRISMA: Preferred Reporting Items for Systematic Reviews and Meta-Analyses

Across the database, we were able to narrow the pool of possible articles down to 1740 articles. After a thorough examination, 71 duplicate articles were eliminated along with 1490 other articles. A total of 179 publications were selected for further review after an initial screening of titles and abstracts, and of these, 178 were assessed followed by the retrieval of complete texts. After determining the eligibility and quality of the full-text papers that had been shortlisted, 33 were selected for the review process. The PRISMA flow chart is given in Figure 1.

We evaluated the results of the risk of bias following the NOS rules [8] and Cochrane Handbook for Systematic Reviews of Interventions [9]. Our results showed that good quality studies predominated, being 51.5% of the total, while the poor and fair quality ones accounted for 33.3% and 15.2%, respectively (Table 3). 

**Table 3:** Risk of Bias Assesment with Newcastle-Ottawa Scale Good quality: 3 or 4 stars in selection domain AND 1 or 2 stars in comparability domain AND 2 or 3 stars in outcome/exposure domain.  Fair quality: 2 stars in selection domain AND 1 or 2 stars in comparability domain AND 2 or 3 stars in outcome/exposure domain.  Poor quality: 0 or 1 star in selection domain OR 0 stars in comparability domain OR 0 or 1 star in outcome/exposure domain [8].

No.	Author	Selection	Comparability	Exposure	Total	Conclusion
1	Miller et al., 2003 [10]​	3	2	3	8	Good quality
2	Li et al., 2005 ​[11]	2	2	3	7	Fair quality
3	Goh et al., 2005 ​[12]	3	2	2	7	Good quality
4	Plestina et al. 2005 ​[13]	2	2	1	5	Poor quality
5	Varghese et al., 2005​ [14]	3	1	3	7	Good quality
6	Krige et al., 2009 ​[15]	3	1	3	7	Good quality
7	Wang et al., 2011 ​[16]​	3	1	0	4	Poor quality
8	Kim et al., 2011 ​[17]	3	1	2	6	Good quality
9	de Souza et al., 2012 [18]	3	1	1	5	Poor quality
10	Hunter and Hamdy, 2013​ [19]​	2	2	1	5	Poor quality
11	Eslam et al., 2013​ [20]​	3	2	3	8	Good quality
12	Xu et al., 2014 [21]	2	1	3	6	Fair quality
13	Yang et al., 2014 [22]	2	2	2	6	Fair quality
14	Zhao et al., 2014 [23]	2	2	3	7	Fair quality
15	Kim et al. 2015 [24]	3	2	1	6	Poor quality
16	Ding et al., 2016 [25]	3	1	1	5	Poor quality
17	Hung et al., 2016 [26]	3	2	3	8	Good quality
18	Kraja et al., 2017 [27]	3	2	3	8	Good quality
19	Giannini et al., 2018 [28]	3	2	3	8	Good quality
20	Zhu et al., 2018 [29]	4	2	2	8	Good quality
21	Ibrahim et al., 2019 [30]	3	1	1	5	Poor quality
22	Xie et al., 2020 ​[31]	4	2	2	8	Good quality
23	Wang et al., 2020 ​[32]	4	2	2	8	Good quality
24	Wang et al., 2020 ​[33]	4	2	2	8	Good quality
25	Salahshour et al., 2020 ​[34]	3	2	2	7	Good quality
26	Lim et al., 2021 ​[35]	4	2	2	8	Good quality
27	Agarwal et al., 2021 ​[36]	2	2	2	6	Fair quality
28	Gao et al., 2021 ​[37]	3	2	1	6	Poor quality
29	Liu et al., 2021 ​[38]	2	2	1	5	Poor quality
30	Saleem et al., 2022 ​[39]	1	1	1	3	Poor quality
31	Asghar et al., 2023 ​[40]	3	1	2	6	Good quality
32	Lou et al., 2023 ​[41]	2	1	1	4	Poor quality
33	Hou et al., 2023 [42]	3	2	2	7	Good quality

The primary outcomes extracted from the finalized studies were risk factors associated with bleeding in esophageal variceal disease in the context of portal hypertension irrespective of the etiology. The included studies were conducted across a broad geographic range, including countries such as China (33.3%), Taiwan (9.1%), South Korea (9.1%), Egypt (9.1%), Spain (6.1%), India (6.1%), Pakistan (6.1%) and in lesser proportions Australia, Albania, Croatia, Italy, Iran, Singapore, South Africa, and USA. We reviewed 27 cohort studies (81.8%) and six case-control studies(18.2%) with a total of 54,065 participants evaluated for risk of bleeding due to various underlying conditions such as cirrhosis from all etiologies (81,8%), hepatitis B cirrhosis (6.1%), hepatocellular carcinoma (HCC) (3%), and chronic hepatitis C infection (3%), alcoholic cirrhosis (3%), and chronic liver disease (3%) (Table 4).

**Table 4:** General outcomes of the included studies CSA: cross-sectional surface area; SEM: Sum of the CSA of esophageal measure; LGV: left gastric vein; PV: portal vein; Tbil: total bilirubin; AVH: acute variceal hemorrhage; HCC: hepatocellular carcinoma; EVB: esophageal variceal bleeding; LSPS: LSM–spleen diameter to platelet ratio score; NSBB: non-selective beta blocker; MELD: Model for End-Stage Liver Disease; HOMA: Homeostasis Model Assessment; IR: insulin resistance; HVPG: hepatic venous pressure gradient; LGVV: left gastric vein velocity; LGVBFD: left gastric vein blood flow direction; MUI: MELD-US-Doppler Index; GEVB: gastro-esophageal variceal bleeding; DM: diabetes mellitus; PVTT: portal vein tumor thrombosis; PVT: portal vein thrombosis; PTVE: percutaneous transhepatic variceal embolization; LSM: liver stiffness measurement; PC: platelet count; GEV: gastroesophageal varices; NPV: negative predictive value; Pl: platelet; kP: kilopond; HR: hazard ratio; AUC: area under the curve; INR: international normalized ratio; EVBL: esophageal variceal band ligation; EVL: esophageal variceal ligation; vEF: Von Willebrand factor; VITRO: vEF antigen/platelet ratio; AUROC: area under the receiver operating characteristics; RCS: red-color sign; VAP: Varices and Portal Hypertension Gastropathy; Plt/S-D: platelet count and spleen diameter ratio; MDCT: multidetector CT; CLD: chronic liver disease; aCLD: advanced CLD; ML: machine learning; SMV: superior mesenteric vein; SPV: splenic vein; AST: aspartate aminotransferase;  RadScore: radiomic score; UGIB: upper gastro-intestinal bleeding; HCT: hematocrit;  ALT: alanine aminotransferase; GGT: gamma glutamyl transferase; NLR: neutrophil-lymphocyte ratio; SGV: short gastric vein; HRV: high-risk varices

No.	Author and Year	Country	Type of Study	Number of patients	Population characteristics	Evaluation	Results	General interpretation
1	Miller et al. (2003)​ [10]	USA	Case-control	28	Portal hypertension and esophageal varices without prior bleeding	Risk of variceal bleeding, based on endoscopic ultrasound	The mean CSA SEM of the sum of the esophageal varices in these patients was 0.77	There is a 76-fold increase per year in the risk of future variceal bleeding for each 1 cm^2^ increase
2	Li et al. (2005)​ [11]​	China	Cohort	120	Esophageal varices but without any previous bleeding	Portal hemodynamics and relationship with size of esophageal varices seen at endoscopy might predict variceal bleeding	Diameter and blood flow velocity of the LGVs were higher for EVBs. Variceal bleeding was more frequent in patients with a diameter of LGV >6 mm. and with a hepatofugal flow velocity >15 cm/s"	Important combination is endoscopic findings followed by the LGV hemodynamics. Duplex-Doppler ultrasonography has no value in the identification of patients with cirrhosis at risk of variceal bleeding. Hemodynamics of the LGV appears to be superior in predicting bleeding.
3	Goh et al. (2005) ​[12]​	Singapore	Cohort	40	Patients with bleeding from esophageal varices confirmed on endoscopy	Criteria of thrombocytopenia, splenomegaly, and ascites were applied to see how accurately they performed in predicting bleeding esophageal varices.	55% had thrombocytopenia, 45% had splenomegaly, and 27.5% had ascites. Six patients had all three criteria. 12 patients with bleeding varices did not have any of the criteria.	Thrombocytopenia, splenomegaly, or ascites are unreliable predictors of bleeding esophageal varices.
4	Plestina et al. (2005) [​13]​	Croatia	Cohort	99	Patients with liver cirrhosis and portal hypertension	Role of Doppler ultrasonography of the portal vein	Patients with variceal red signs had significantly higher values of portal diameter, cross-sectional area, blood flow volume and congestion index, while the perfusion pressure gradient and the platelet/spleen ratio were lower.	Color Doppler ultrasonography is a useful non-invasive method for evaluating the risk of esophageal variceal bleeding in patients with liver cirrhosis.
5	Varghese et al. (2008) [14]​	India	Cohort	205	Patients with liver cirrhosis with esophageal varices	Determine the predictors of first and subsequent bleed.	Grades Ill and IV esophageal varices, presence of cherry-red spots and fundal varices were significant risk factors for index bleed and rebleed.	Higher grades of varices, presence of cherry-red spots and fundal varices predicted variceal bleed.
6	Krige et al. (2009) [15]	South Africa	Cohort	310	Alcoholic cirrhotic patients with AVH who underwent endoscopic variceal injection treatments	Bleeding esophageal varices found were treated with injection sclerotherapy	>6 units of blood transfused, the presence of ascites, and a bilirubin level >51 mmol/l were the best set of significant predictor covariates. In the multivariate logistic regression analysis model, bilirubin levels greater than 51 mmol/l and >6 units of blood transfused during the initial hospital admission were significant predictors of rebleeding.	Bilirubin levels >51 mmol/l and transfusion of >6 units of blood were predictors of variceal rebleeding.
7	Wang et al. (2011) ​[16]​	China	Case-control	186	Patients with cirrhosis with no HCC or malignant diseases and endoscopy examination with bleeding from esophageal varices	Documentation of clinical, biochemical, and treatment methods that might contribute to variceal rebleeding.	Multivariate stepwise regression analysis showed positively correlations with rebleeding: Child-Pugh grade B, Tbil, creatinine and the cumulative volume of blood transfusion. The presence of ascites and prophylactic antibiotics were negatively correlated	Rebleeding in cirrhotic inpatients was associated with more blood transfusions, Child-Pugh grade B, higher Tbil and creatinine.
8	Kim et al. (2011) [17]​	South Korea	Cohort	577	B-viral cirrhosis patients none of whom experienced EVB.	Assess risk of EVB using the LSPS model.	Multivariate analysis found higher LSPS a significant predictor, alongside large variceal sizes and Child-Pugh classifications B/C.	LSPS is a reliable predictor for EVB risk.
9	de Souza et al. (2012) [18]	Spain	Case-control	89	Cirrhotic patients with acute esophageal variceal bleeding; no HCC	Compare the risk of rebleed between patients taking prophylactic B-Blockers and controls, after treatment with band ligation and B-Blockers post bleed.	Episode of bleeding while on NSBBs had further bleeding, compared with controls. Primary prophylaxis with NSBBs was an independent predictor.	Patients who have their first episode of variceal bleeding while on primary prophylaxis with a b-blocker agent have an increased risk of further bleeding
10	Hunter and Hamdy (2013) ​[19]​	Egypt	Cohort	100	Patients presenting with hematemesis and/or melena due to bleeding varices.	Determine the risk factors for re-bleeding within five days and mortality up to six weeks in patients with cirrhosis and acute variceal hemorrhage	Group I: patients who survived >6 weeks after endoscopic management and did not rebleed. Group II: patients who died <6 weeks of AVH. Group III: patients who rebled or died within five days of AVH. MELD score was significantly higher in group II and group III as active bleeding at time of endoscopy was present in 8% of group I, 70% of group II and 53.3% of group III, while white nipple sign was present in 10.6% of group I, 90% of group II and 73.3% of group III.	High MELD score (>18), presence of active bleeding or white nipple sign at time of endoscopy are significant predictors for early rebleeding after AVH.
11	Eslam et al. (2013) ​[20]​	Spain and Egypt	Cohort	357	Cirrhotic patients without previous variceal bleeding	Assess the involvement of metabolic factors in the prediction of portal hypertension, esophageal varices and risk of variceal bleeding in cirrhotic patients.	Esophageal varices was independently associated with lower platelet count, raised HOMA index and adiponectin levels. This extended to subset analysis in patients with Child A cirrhosis. HOMA index and adiponectin levels significantly correlated with HVPG. Beside Child-Pugh class, variceal size, glucagonemia, HOMA index were associated with higher risk of variceal bleeding.	HOMA score correlates with HVPG and independently predict clinical outcomes. Platelet count, IR assessed by HOMA-IR and adiponectin significantly predict the presence of esophageal varices in cirrhotic patients.
12	Xu et al. (2014) [21]	China	Cohort	486	Patients with decompensated cirrhosis	Evaluate risk of EVB in decompensated cirrhosis patients based on US-Doppler to assess LGVV and LGVBFD	MUI, a new index, was developed; when the MUI was set at 46, the index had high diagnostic accuracy (85.8%), with high specificity (80%) and sensitivity (87.27%).	The MUI, a noninvasive and affordable index, can predict EVB occurrence in decompensated cirrhotic patients and serve as an alternative to conventional methods
13	Yang et al. (2014) ​[22]	Taiwan	Case-control	146	Patients with liver cirrhosis and no HCC	Elucidate whether DM is an independent risk factor for GEVB among cirrhotic patients.	Patients with DM had higher ratio of Child-Pugh Class B/C, renal insufficiency, and history of GEVB. GEVB was associated with Child-Pugh Class B/C, ascites, hepatic encephalopathy, and low platelet counts. Multiple logistic regression analysis, Child-Pugh class B/C and DM were identified as independent predictors of GEVB. In the subgroup analysis, DM correlated with GEVB in patients with Child-Pugh Class A but not in Class B/C	DM is independently associated with GEVB in cirrhotic patients, especially in those with Child-Pugh Class A.
14	Zhao et al. (2014) ​[23]​	China	Cohort	101	Patients with liver cirrhosis and AVH without concomitant HCC.	Determine the risk factors for six-week rebleeding after percutaneous transhepatic variceal embolization.	In multi-variable analyses high-risk stigmata of variceal bleeding, the obliteration range of PTVE, and an HVPG ≥ 20 mmHg were significantly associated with the risk of rebleeding	High-risk stigmata, PTVE with trunk obliteration, and a hepatic vein pressure gradient are predictive factors for six-week rebleed episode.
15	Kim et al. (2015) ​[24]	Korea	Cohort	125	Cirrhotic patients without variceal bleeding, or intrahepatic malignancy.	Evaluate the association of liver stiffness and SS as measured by ARFI, with the presence and severity of esophageal varices and variceal hemorrhage	SS was significantly higher in patients with varices than in those without varices. A tendency toward increasing SS levels was observed with increasing severity of varices and variceal hemorrhage.	SS was significantly correlated with the presence, severity, and bleeding risk of esophageal varices.
16	Ding et al. (2016) ​[25]​	Australia	Cohort	271	Compensated cirrhotic patients, no HCC, no Portal HT.	Develop a simple clinical rule to exclude the presence of high-risk GEV in patients with Child–Pugh A cirrhosis using LSM ± platelet count.	combined model based on LSM and PC was more accurate for excluding the presence of high-risk GOV than either alone, with the combination of LSM ≤25 kPa and Pl ≥100 having an NPV of 100% in both the training and validation cohorts.	The combination of LSM ≤25 kPa and Pl ≥100 can be used in clinical practice to exclude the presence of high-risk GEV in patients with Child-Pugh A cirrhosis.
17	Hung et al. (2016) ​[26]​	Taiwan	Cohort	38172	Cirrhotic patients without previous EVB	Determine the cumulative incidence of EVB among cirrhotic patients and identify the predictors of EVB occurrence.	Regression analysis showed that the HR of EVB history was 4.42 for EVB occurrence. Other predictors for EVB occurrence included hepatocellular carcinoma, young age, ascites, alcohol-related disorders, peptic ulcer bleeding and diabetes mellitus.	HCC, young age, ascites, alcohol-related disorders, PUB, diabetes mellitus and a history of EVB were factors found to be associated with increased risk of EVB occurrence. Cirrhotic patients have a fourfold increased risk of future EVB following the first incidence of EVB.
18	Kraja et al. (2017) [​27]​	Albania	Cohort	139	Cirrhotic patients without previous bleeding, no HCC, No Hepatitis B or C	Assess predictors of EV and variceal bleeding using non-invasive markers.	A cut-off value of 3.23 for FIB-4 was a significant predictor of esophageal varices, with a sensitivity of 72%, a specificity of 58% and a proportion of AUC of 66%.	Despite the low diagnostic accuracy, FIB-4 appears the most efficient non-invasive liver fibrosis marker which can be used as an initial screening tool for cirrhotic patients.
19	Giannini et al. (2018) ​[28]​	Italy	Cohort	109	Patients with cirrhosis and thrombocytopenia with no prior bleeding	Evaluate factors predictive of post-EVBL bleeding in cirrhotic patients with thrombocytopenia	INR and platelet counts were not predictors of post-EVBL bleeding. A fibrinogen cut-off of 179 mg/dL had 98.6% negative predictive value for bleeding.	Low fibrinogen levels are associated with an increased risk of bleeding following prophylactic EVBL in cirrhotic patients and may be used to stratify patients' risk.
20	Zhu et al. (2018) ​[29]​	China	Cohort	88	Patients with HBV-related cirrhosis without prior bleeding episode.	Analyze the diagnostic accuracy of LSM for predicting esophageal variceal grading and risk of EVB in cirrhosis.	Esophageal varices grade was highly correlated with LSM and the LSPS in cirrhosis. Meanwhile, LSM and spleen diameter were two independent factors for predicting EVB.	LSM and spleen diameter had excellent abilities to predict EVB.
21	Ibrahim et al. (2018) [30]​	Egypt	Case-control	130	Patients with hepatitis C-related liver cirrhosis.	Explore the vEF and VITRO score in the prediction of variceal bleeding in patients with portal hypertension	The mean levels of the vEF antigen and the VITRO score were higher in patients with variceal bleeding. Levels of vEF were correlated positively with esophageal varices grade.	Serum vEF level and the VITRO score are potential noninvasive biomarkers for the prediction and risk stratification of variceal bleeding in hepatitis C-related liver cirrhosis.
22	Xie et al. (2020) [​31]​	China	Cohort	264	Patients with cirrhosis and portal hypertension.	Evaluate the risk of first upper gastrointestinal bleeding by CT for esophageal varices	The diameter of esophageal varices was 7.83±2.76mm in bleeding group, and 6.57±3.42mm in non-bleeding group. The number of venous vessels was 4.5±2 in bleeding group, whereas being 4±2 in non-bleeding group. The blood vessel area was 1.73±1.15 cm2 in bleeding group, and 1.12±0.89 cm2	Among all three indicators of the total area, diameter, and number of sections of the esophageal varices, the total area of esophageal varices showed more accuracy as a potential and novel indicator for bleeding prediction.
23	Wang et al. (2020) [​32]​	Taiwan	Cohort	7084	Patients with cirrhosis and EV, grade 1 or minimal, and no RCS or no red wale marks, no prior bleeding.	Investigate if the Fibrosis Index marker could predict subsequent EVB	The FI with cut-off value of 3.95 showed an NPV of 94.3% and an AUROC of 62.95% for predicting subsequent EVB within 1 year.	Low FI scores showed a high NPV and moderate AUROC in predicting subsequent EVB, signifying clinically non-significant portal hypertension.
24	Wang et al. (2020) [​33]​	China	Cohort	189	Patients with cirrhosis and no prior bleeding episode from esophageal varices	Determine the accuracy of two kinds of scoring models in predicting the degree of esophageal varices and EVB: VAP and Plt/S-D	The AUROC of the VAP score model and Plt/S-D score model were 0.901 and 0.835, respectively.	These two kinds of scoring models can predict the degree of esophageal varices and bleeding in liver cirrhosis patients and have good predictive accuracy.
25	Salahshour et al. (2020) ​[34]​	Iran	Cohort	124	Cirrhotic patients who had undergone both UGIT endoscopy and contrast-enhanced MDCT	Investigate the associated MDCT features for esophageal varices and EVH	Findings of esophageal varices in MDCT analysis were the best predictor of esophageal varices or EVH, and presence (and/or size) of specific collaterals showed a significant relationship with both esophageal varices and EVH: coronary, short gastric and paraesophageal.	The presence and size of various collaterals are related to presence of esophageal varices and higher risk of EVH.
26	Lim et al. (2021) ​[35]​	Korea	Cohort	1709	Patients with HCC without any cancer-related symptoms without prior variceal hemorrhage	Association of PVTT in the incidence of esophageal varices and VB in HCC and determine the indications for variceal screening and prophylaxis.	Prolonged prothrombin time, lower platelet count, presence of extrahepatic metastasis, and Vp4 PVTT were independent risk factors related to high-risk varices. Presence of high-risk varices and sorafenib use for HCC treatment were significant predictors of variceal bleeding in the complete set of patients with PVTT	PVTT increases the incidence for HRV and variceal bleeding. PT, low platelet count, presence of extrahepatic metastasis, and Vp4 PVTT are risk factors for HRV. HRV and sorafenib increases the risk of VB in HCC
27	Agarwal et al. (2021) [36]​	India	Cohort	828	Patients with cACLD with esophageal varices	Assess if ML could be used for predicting future VB more accurately.	The accuracy of ML-based model to predict future VB was 98.7%, 93.7 %, and 85.7 % in derivation, internal validation, and external validation cohorts, respectively, which was better than endoscopic classification [58.9%] alone.	Application of ML model improved the performance of endoscopic stratification to predict VB in patients with cACLD with EVs.
28	Gao et al. (2021) [​37]​	China	Case-control	218	Cirrhotic patients with previous VB and treatment with EVL and/or gastric variceal obturation"	Determine the effect of PVT on rebleeding in patients with AVB after EVL	Patients with PVT had a higher rate of 14-day and 6-week rebleeding. The Child–Pugh class, PVT, albumin<30 g/L, and number of bands were identified as the predictors for 14-day rebleeding; multivariate analysis revealed only the number of bands as independent factor. PVT and albumin<30 g/L were identified as predictors for 6-week rebleeding; only PVT was found to be the independent factor in multivariate analysis. Further analysis showed that SMV thrombosis is the only risk factor predicting six-week rebleeding in patients with PVT	PVT was associated with high 14-day and six-week rebleeding in patients after EVL. SMV thrombosis was the only risk factor for six-week rebleeding in patients with PVT. High albumin levels may serve as a protective factor for the 14-day and six-week rebleeding risk.
29	Liu et al. (2021) ​[38]​	China	Cohort	317	Patients with cirrhotic esophageal varices, No HCC or PVT	Develop a non-invasive prediction model for the risk of EVB using dual-energy CT.	Diameter of esophageal varices, diameter of SP, ascites, iodine concentration in SGV, iodine concentration in spleen were independent predictors of EVB risk.	Combination of dual-energy CT with conventional CT may have added value for non-invasive prediction of EVB compared to conventional CT.
30	Saleem et al. (2022) [​39]​	Pakistan	Cohort	50	Patients with CLD who previously had evidence of varices and had at least one episode of rebleeding after EVBL	Explore the predictors of rebleeding in chronic liver disease	Rebleeding was significantly associated with grade of varices, presence of red sign on upper GI endoscopy, site of varices, splenic size and coagulopathy.	Rebleeding in CLD patients following EVBL is predicted by grade, extent and site of varices, red sign on upper GI endoscopy, splenic size and coagulation disturbances.
31	Asghar et al. (2023) [​40]​	Pakistan	Cohort	93	Patients with active esophageal variceal bleeding	Determine predictors of re-bleeding after esophageal variceal banding	The major contributing factors to re-bleeding were the severity of cirrhosis, grades and columns of EVs, number of bands ligation and findings of red wale sign. Increasing age and duration of cirrhosis were contributing predictors of increased re-bleeding risk.	Predictive factors for rebleeding: severity of cirrhosis, grades and columns of esophageal varices, number of bands ligation and findings of red wale sign
32	Lou et al. (2023) ​[41]​	China	Cohort	211	Patients with cirrhosis	Develop a nomogram based on clinical variables and radiomics to facilitate the noninvasive prediction of EGVB in cirrhotic patients.	Albumin, fibrinogen, PVT, AST, and spleen thickness were selected as independent clinical predictors of EGVB. RadScore, constructed with five CT features of the liver region and three of the spleen regions, performed well. There was excellent predictive performance for the clinical-radiomics model, comparing with the existing noninvasive models such as AST/PC ratio and Fib-4 scores, the combined model had better predictive accuracy.	The clinical-radiomics nomogram can noninvasively predict whether cirrhotic patients will develop EGVB
33	Hou et al. (2023) [42]	China	Cohort	1100	Patients with cirrhosis.	Estimate the EGVB risk in patients with liver cirrhosis using an artificial neural network.	A total of 12 independent risk factors, including gender, drinking and smoking history, decompensation, ascites, location and size of varices, ALT, GGT, HCT and NLR levels as well as RBC count were evaluated and used to establish the ANN model, which estimated the 1-year EGVB risk. The ANN model had an AUC of 0.959	The ANN model accurately predicted the one-year risk for EGVB in liver cirrhosis patients and might be used as a basis for risk-based EGVB surveillance strategies.

The included studies found the following to be predictors for esophageal variceal bleeding: (i) predictive scores, models, and indexes (48.5%); among them, the most frequent were Child-Pugh class B/C (12.1%) and Fib-4 (6.1%); (ii) imaging factors (33.3%) including liver stiffness measurement, spleen size, diameter or stiffness, total area of esophageal varice, hemodynamics of the left gastric vein, hepatic vein, and portal vein, portal vein thrombosis (PVT)/superior mesenteric vein thrombosis, ascites, iodine concentration in the short gastric vein and spleen, and the presence and size of collaterals; (iii) endoscopic factors (24.2%) such as red wale sign, large varices size, higher grade of varices and number of bands after esophageal variceal ligation; (iv) laboratory factors (excluding platelet count) (18.2%) like high bilirubin, high creatinine, high aspartate aminotransferase (AST) levels, coagulation parameters, low albumin and low fibrinogen; and (v) thrombocytopenia (9.1%). Other factors were diabetes mellitus (6.1%), the use of sorafenib in HCC (3%) and prophylactic nonselective beta‐blocker (NSBB) in cirrhosis (3%). It is important to state the predictive scores, models and indexes used many parameters including some of the previously mentioned factors and others like: alanine transaminase (ALT), gamma-glutamyl transferase (GGT), RBC, CT features of the liver and spleen, glucagonemia and interventional radiology (IR), age, alcohol and smoking history (Table 4). 

Discussion

Predictive Factors

The Child-Pugh score, evaluated across multiple articles [​20,22,24,37], plays a significant role in assessing the risk of bleeding in cirrhotic inpatients. Specifically, rebleeding in cirrhotic inpatients was associated with Child-Pugh grade B [16]. The Child-Pugh score is a well-established system used to evaluate the severity of cirrhosis, with Grade B indicating a moderate degree of liver dysfunction. In the context of variceal bleeding risk, this grade signifies a higher risk of rebleeding. 

The Fibrosis Index stands out as a genuinely predictive factor, and what's even more reassuring is that the studies investigating it were of high quality. The Fibrosis index with a cut-off value of 3.95 showed a negative predictive value (NPV) of 94.3% and an area under the receiver operating characteristic (ROC) curve (AUROC) of 62.95% for predicting f within one year [32]. FIB-4 appears to be the most efficient non-invasive liver fibrosis marker which can be used as an initial screening tool for cirrhotic patients [27].  

Ultrasound, Endoscopy, and Other Imaging Values

Predictive factors for rebleeding identified through endoscopy encompassed specific findings, which included the presence of the red wale sign or cherry red spot, esophageal variceal grading, and the number of band ligations performed [14,40,39]. These factors were consistently supported across multiple articles, and their reliability was reinforced by their low risk of bias. 

Ultrasound analysis revealed significant associations, such as the assessment of left gastric vein hemodynamics. Notably, EVB was more frequently observed in patients with a left gastric vein diameter exceeding 6 mm and a hepatofugal flow velocity surpassing 15 cm/s [11]. Moreover, a comprehensive MUI (Model for End-Stage Liver Disease (MELD)-Ultrasound Doppler index), which factored in the assessment of the left gastric vein, emerged as another predictive factor [21]. While both articles exhibited a fair quality concerning the risk of bias, the consensus drawn from their findings underscores the strength of these factors as reliable predictors. 

High liver stiffness, as determined through ultrasound evaluation, has been established as a reliable predictor of bleeding risk [17]. This finding is further corroborated by an additional study indicating that low liver stiffness measurements effectively excluded patients with high-risk varices, particularly in individuals with Child-Pugh A cirrhosis [25]. In contrast, spleen stiffness demonstrates a correlation with the presence, severity, and bleeding risk of esophageal varices, as indicated in one study; however, it’s worth noting that this study was of suboptimal quality due to a risk of bias [24]. 

Qingjing Zhu et al. present strong evidence that both spleen and liver stiffness are excellent predictors of liver stiffness measurement and spleen diameter, with the study’s quality being good [29]. Nevertheless, it’s crucial to recognize that these factors are interconnected and not necessarily independent of each other, limiting the findings of the spleen diameter as a predictor. This interrelation suggests that the outcome of one factor might be influenced by another. In summary, liver stiffness is a reliable predictor, while spleen diameter requires further research and exploration to fully understand its predictive capabilities. 

Another factor evaluated by ultrasound was the portal vein diameter. Patients with variceal red signs had significantly higher values of portal diameter, cross-sectional area, blood flow volume, and congestion index [13]. The high risk of bias in the study limits the interpretation. Hepatic vein pressure gradient is a predictive factor for six-week rebleed episode [23].  

Portal vein tumor thrombosis (PVTT) elevates the incidence of high-risk varices and EVB [35]. This underscores the clinical significance of PVTT in patients, particularly those with HCC, in terms of the associated risk of these adverse outcomes. Gao et al. further substantiate this by indicating that patients with PVTT exhibit a higher rate of rebleeding, both at the 14-day and six-week intervals [37]. This reinforces the idea that PVTT contributes to the risk of variceal bleeding recurrence. 

The utilization of CT scans as an imaging tool in assessing esophageal varices and their potential to predict bleeding events have been the subject of recent investigations, notably in high-quality studies: the total area of esophageal varices showed more accuracy (being 7.83±2.76mm in bleeding group) as a potential and novel indicator for bleeding prediction [31]. Findings of esophageal varices in multidetector CT (MDCT) analysis were the best predictor of esophageal varices or esophageal variceal hemorrhage, and the presence (and/or size) of specific collaterals showed a significant relationship with both esophageal varices and esophageal variceal hemorrhage: coronary, short gastric, and paraesophageal [34]. These particular findings not only provide a novel indicator for predicting bleeding but also underscore the potential of CT scans to offer precise assessments of esophageal varices, enhancing clinical decision-making. Therefore, it is advisable that CT scans be integrated more widely into the clinical management of patients with esophageal varices, particularly in the context of risk assessment and preventive measures. 

Another imaging measurement provided by the analysis of a dual-energy CT scan was the iodine concentration in the short gastric vein and in the spleen [38], and although they were found to be independent predictors for EVB risk, usefulness as a predictor of EVB is limited due to a high risk of bias of the study. 

Ascites

Ascites was determined to be a risk factor for variceal bleeding according to the findings of Krige et al. [15], Yang et al. [22], and Hung et al. [26], two of them being high-quality studies. Goh et al.'s study, which had low risk of bias, determine thrombocytopenia, splenomegaly, or ascites as unreliable predictors of bleeding esophageal varices [12]. It is interesting to notice that only 27.5% of patients evaluated in this study had ascites. Only six patients had the three criteria, so we could consider that the sample size was relatively small to conclude that ascites is not related to the risk of bleeding. Wang et al. [16] determined that ascites was negatively correlated to EVB, although this study had a high risk of bias, therefore being less significative than the previous ones stating the opposite. 

Platelets 

Our comprehensive review of high-quality studies reveals a consistent consensus that questions the utility of thrombocytopenia, splenomegaly, and ascites as reliable predictors of EVB in patients with liver cirrhosis. Goh et al. emphasize the unreliability of thrombocytopenia, splenomegaly, and ascites in predicting EVB [12]. This challenges the traditional view that low platelet count indicates EVB risk, highlighting the need for a reevaluation of the factors contributing to EVB. Giannini et al. support the notion that platelet counts are not suitable predictors for post-endoscopic variceal band ligation (EVBL) bleeding [28]. These findings suggest the limitations of these commonly considered parameters in assessing EVB risk. 

Laboratory Values

Low fibrinogen levels are associated with an increased risk of bleeding following prophylactic EVBL in cirrhotic patients and may be used to stratify patients' risk [28]. Another coagulation parameter considered to be a predictor of variceal bleeding is the Von Willebrand Factor (vEF) and the VITRO (vEF antigen/ platelet count) score [30], although our analysis determined this study to have a high risk of bias, therefore being limited in its application in predicting bleeding from esophageal varices. 

Albumin was introduced as a variable considered in clinical radiomics nomogram to predict EVB in cirrhotic patients [41]. It’s important to highlight the study’s high risk of bias. PVT and low albumin levels <30 g/l were identified as potential predictors for six-week rebleeding following EVBL [37]. However, further analysis revealed that only PVT remained as an independent factor and albumin did not retain its predictive power in this context. The presence of PVT in cirrhotic patients often complicates the clinical picture, and it can be a crucial factor in assessing rebleeding risk. However, the limitations of the high risk of bias in this study should be taken into account, and further investigations are necessary to confirm these results. 

The role of high creatinine levels in predicting rebleeding in cirrhotic inpatients has been examined by Wang et al. [16]. However, their study's quality does not meet the criteria required for robust predictive factors. As a result, the reliability of high creatinine as an independent predictor for rebleeding remains questionable. In a study by Luo et al., AST levels were considered part of a clinical radiomics nomogram thar can noninvasively predict whether cirrhotic patients will develop EVB [41]. However, it’s important to notice the quality of their study was poor and there was simultaneous evaluation of albumin, fibrinogen, PVT, AST, and spleen thickness. Further research is needed to validate the findings and assess the generalizability of the clinical-radiomics model. Future studies should also explore the feasibility of implementing this nomogram in clinical practice. 

Bilirubin levels were also considered by two different studies [15,16], and both found that high bilirubin levels are directly correlated to rebleeding of esophageal varices. However, it is important to take into account that one of them has a high risk of bias, needing further investigation on this parameter. 

Other Predictive Factors

Diabetes mellitus: Diabetes mellitus has been considered a potential predictive factor for bleeding, especially in those with Child-Pugh Class A cirrhosis, as indicated in a study by Yang et al. [22], with a fair quality in terms of potential bias. Notably, it's intriguing to observe that a higher percentage of patients with diabetes were in Child-Pugh Class B/C, experiencing renal insufficiency, and having a history of gastroesophageal variceal bleeding. However, it's essential to acknowledge that the the outcomes of Yang et al.'s study might have been influenced by the specific characteristics of the population under investigation. Furthermore, the study was constrained by a relatively small number of patients. Diabetes was also a subject of evaluation in a separate study, which also examined age, ascites, and alcohol-related disorders as potential factors, and this study was of good quality [25]. However, the need for further research in this area remains, given the complex interplay of factors and potential limitations in the existing studies. 

Beta-blockers: According to Kim et al., patients who had their first episode of variceal bleeding while on primary prophylaxis with a b-blocking agent have an increased risk of further bleeding [17]. However, their study had a poor quality and there is no support from other studies.

Protective Factor

High albumin levels were stated to serve as a protective factor by Gao et al. [37], but the high risk of bias of their study subjects this factor to further evaluation in the future. No further studies found protective factors for EVB.

Limitations

It is essential to recognize that existing research has limitations, including varied treatment protocols and potential biases. The review adhered to its inclusion criteria for patients with hepatic cirrhosis of all etiologies; however, some studied patients were exposed to cirrhosis of infectious causes, primarily hepatitis B or C, and of HCC in a compensated state. These characteristics could potentially have influenced our findings. It is noteworthy that our research was limited to a specific number of academic databases, focused on articles published in English and Spanish, and was based on a specific time frame. As a result, we understand that there may be a potential bias due to these limitations that defined our search methodology. Therefore, there is a risk of omission of older studies and relevant research in different languages. The possibility that literature that is under review or in the editing process for publication may have been missed is also something to consider. Despite our dedicated search methodology and efforts to include worldwide studies, Asian regions predominated, which could influence our conclusion. It is possible that other publications may have escaped our attention and analysis. It is important to highlight that this is an exceptional systematic review on this topic, for which there were no previous peers, providing essential information that could bridge potential gaps in knowledge. 

## Conclusions

This comprehensive systematic review has explored a wide range of factors associated with the risk of EVB in patients with portal hypertension. It has shed light on various predictors, both clinical and laboratory, that have been identified in the literature, providing valuable insights into this critical medical issue. The primary findings suggest that the Child-Pugh score, Fibrosis Index, specific endoscopic findings, ultrasound parameters, portal vein diameter, presence and size of collaterals, CT scan findings, ascites, platelet counts, coagulation parameters, albumin levels, Von Willebrand factor, bilirubin levels, diabetes mellitus, and the use of beta-blocking agents in primary prophylaxis are potential predictive factors for EVB. These findings provide a comprehensive overview of the various aspects that can influence the risk of bleeding in patients with portal hypertension. 

However, it is important to note that some of the identified factors exhibit varying degrees of reliability and may be subject to limitations and biases, as highlighted in the discussion. The quality of the studies and the potential for bias in some cases warrant caution in interpreting these findings. Further research is needed to refine and validate these predictive factors, taking into consideration the potential limitations of the existing studies. In addition, it is essential to acknowledge that the complex interplay of factors and the diversity of patient populations may influence the predictive capabilities of these factors. Therefore, a more tailored and individualized approach to risk assessment for EVB is necessary, accounting for specific patient characteristics and clinical context. 
